# Investigation of *Salmonella pullorum* and *Mycoplasma gallisepticum* by rapid plate serology in parrots victims of wildlife trafficking in the state of Paraíba

**DOI:** 10.3389/fvets.2026.1886000

**Published:** 2026-07-08

**Authors:** Audisio Alves da Costa Filho, Glenisson Ferreira Dias, Adalberto de Menezes Melo Neto, José Romulo Soares dos Santos, Lucas Rannier Ribeiro Antonino Carvalho, Arthur Willian de Lima Brasil

**Affiliations:** 1 Federal University of Paraíba, João Pessoa, Paraíba, Brazil; 2 Department of Wildlife Management, Superintendence of Environmntal Administration of the State of Paraíba (SUDEMA), João Pessoa, Paraíba, Brazil; 3 Department of Physiology & Pharmacology, Karolinska Institutet, Stockholm, Sweden

**Keywords:** *Amazona* spp, *Mycoplasma gallisepticum*, *Salmonella pullorum*, seroreactivity, wildlife trafficking

## Abstract

**Introduction:**

Wildlife trafficking exposes parrots to stressful conditions that favor the transmission of infectious pathogens, including *Salmonella pullorum* and *Mycoplasma gallisepticum*. This study aimed to investigate seroreactivity against these pathogens and to evaluate the associated clinical, morphological, biochemical, metabolic, and hematological alterations in *Amazona aestiva* and *Amazona amazonica* parrots rescued from wildlife trafficking.

**Methods:**

Twenty-nine parrots rescued from wildlife trafficking were subjected to physical examination, morphological evaluation, hematological and biochemical analyses, and Rapid Serum Agglutination (RSA) testing for antibodies against *M. gallisepticum* and *S. pullorum*. Statistical comparisons were performed between reactive and non-reactive groups to identify laboratory alterations associated with seroreactivity.

**Results:**

Seroreactivity to *M. gallisepticum* was observed in 21.73% of A. aestiva and 14.3% of A. amazonica. For S. pullorum, 36.4% (8/22; 95% CI: 16.3–56.6%) of A. aestiva and 28.6% (2/7; 95% CI: 3.7–71.0%) of A. amazonica tested positive. Birds reactive to *S. pullorum* exhibited significantly lower total protein concentrations, whereas *M. gallisepticum*-reactive birds showed significantly lower leukocyte counts and higher glucose concentrations. Significant differences were also observed in calcium and sodium concentrations among groups. Reactive birds presented clinical alterations compatible with gastrointestinal and respiratory involvement, as well as changes in body condition and feather quality.

**Discussion:**

The findings indicate that parrots rescued from wildlife trafficking and reactive to *S. pullorum* or *M. gallisepticum* may exhibit important clinical and laboratory alterations suggestive of multisystemic impairment. As the RSA test detects circulating agglutinating antibodies without distinguishing immunoglobulin classes or confirming active infection, positive results should be interpreted as evidence of previous exposure and immune response rather than current infection. Nevertheless, the findings reinforce the importance of sanitary surveillance, screening programs, and appropriate clinical management in wildlife rehabilitation centers to support animal health, conservation efforts, and prevention of pathogen dissemination.

## 1 Introduction

Wildlife trafficking is the third largest illegal activity worldwide and mainly involves the commercialization of native fauna. This practice is especially common in socioeconomically vulnerable regions, where the use of wildlife products and bushmeat contributes to local subsistence and regional markets (1, 2, 42).

In Brazil, birds represent approximately 75% of the animals admitted to wildlife screening centers, with parrots of the genus *Amazona* spp. being among the most affected. Conditions associated with wildlife trafficking, such as overcrowding, stressful transportation, inadequate confinement, and poor sanitary management, increase susceptibility to infectious diseases (42).

Opportunistic pathogens frequently exploit immunosuppression caused by stress and poor welfare conditions. Among the main bacterial agents associated with trafficked birds are *Salmonella pullorum* and *Mycoplasma gallisepticum*, both of which affect birds and humans and present important zoonotic potential (3–7).

*Salmonella pullorum* is a Gram-negative bacterium commonly associated with enteric diseases and transmitted through contaminated feces, water, or food (8, 9). In parrots, severe enteritis, pneumonia, and infectious hepatitis have been reported. Likewise, *Mycoplasma gallisepticum*, a bacterium lacking a defined cell wall, is mainly transmitted through respiratory secretions and contaminated feces, causing chronic respiratory disease and systemic impairment in infected birds (10, 11).

These pathogens are closely linked to environmental degradation and wildlife trafficking, factors that contribute to the emergence and spread of zoonotic diseases under the One Health perspective (12). Therefore, this study aimed to evaluate infections caused by *S. pullorum* and *M. gallisepticum* in *Amazona aestiva* and *Amazona amazonica* parrots rescued from wildlife trafficking, seeking to better understand the epidemiological and sanitary implications associated with these pathogens.

## 2 Methods

### 2.1 Animals and management

The present study was conducted at the Wildlife Screening Center of the State of Paraíba (CETAS-PB), located in the municipality of Cabedelo, Paraíba, Brazil, where blood aliquots were collected from 22 adult *Amazona aestiva* (*A.Ae*/*A. aestiva*) and seven adult *Amazona amazonica* (*A.Am*/*A. amazônica*) individuals of undetermined sex. The animals were admitted through environmental authorities or voluntary surrender, under authorization of CEUA No. 6752170325 and SISBIO No. 97020.

### 2.2 Restraint, evaluation, and animal screening

Animal restraint was performed according to the procedures described by Cubas et al. (13). The birds were removed from their enclosures and transported to the CETAS-PB clinical facility, where sedation was induced using 2% Isoflurane or 2% Sevoflurane (14). Physical examinations were conducted, recording common name, scientific name, and identification band number. The following parameters were evaluated: body weight; body condition score (1—cachectic, 2—thin, 3—ideal condition, 4—overweight, and 5—obese); keel muscle condition [1—flaccid (animals with no movement), 2—strong (animals with flight or locomotion habits)]; body fat deposition (1—absence of fat, 2—fat islands, 3—fat archipelago, 4—apparent fat deposition, and 5—high fat accumulation); feather coloration [1—normal, 2—darkened, 3—color alteration (yellowish or greenish)] in the wings, back, and chest regions. Hydration status, fecal appearance (normal or darkened), and the presence of respiratory wheezing were also evaluated.

### 2.3 Blood collection, hematology, and serum biochemistry

**Figure 1:**
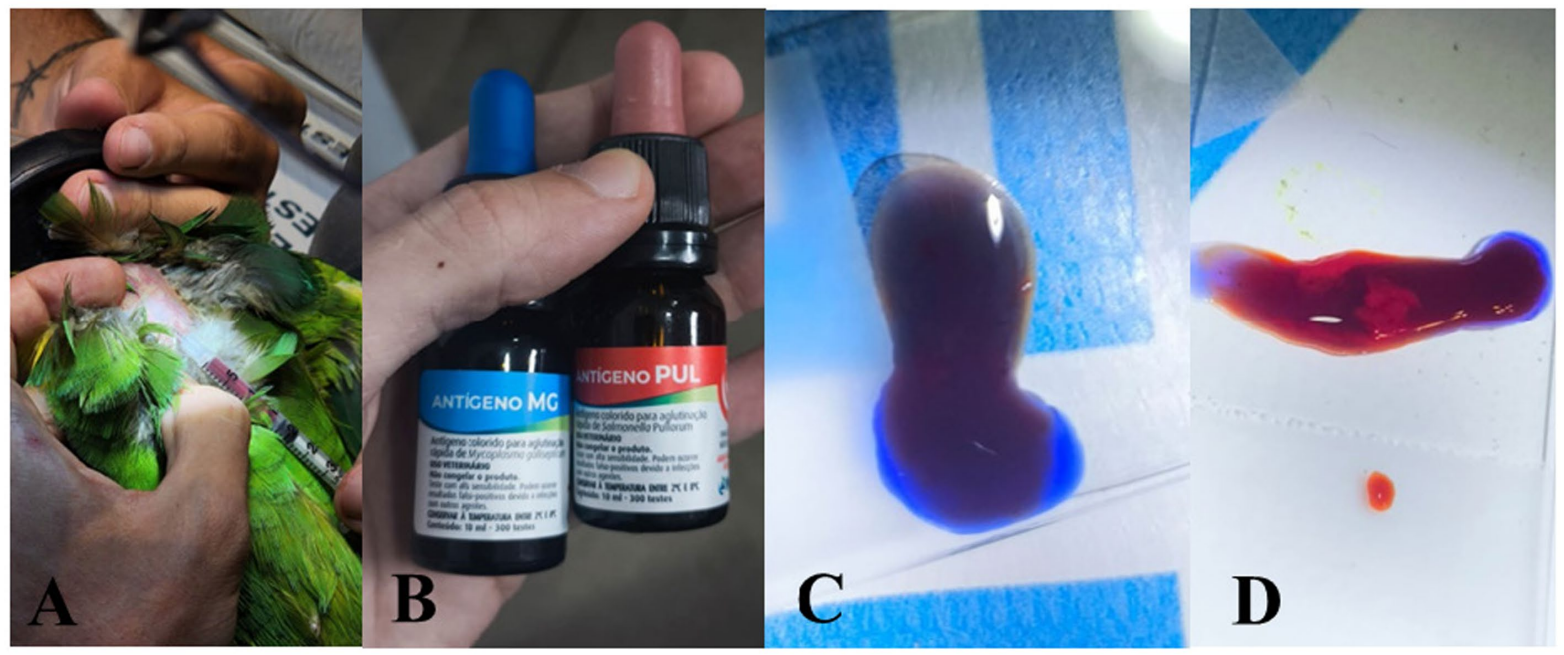
**(A)** Jugular blood collection from *A. aestiva* rescued from wildlife trafficking. **(B)** Bottles containing the antigens Pulor Test® and Myco-Galli Test®. **(C)** Non-reactive blood aliquot from *A. aestiva* rescued from wildlife trafficking. **(D)** Agglutinated blood aliquot from *A. aestiva* rescued from wildlife trafficking.

Venous blood samples with anticoagulant (EDTA) were collected, respecting the limit corresponding to 10% of the animal’s live body weight, for hematological and biochemical analyses. Blood collection was performed through puncture of the jugular or ulnar always considering hydration status and physical examination findings (Figure 1A).

Hematological analyses were performed using the Rosenfeld staining technique. Hematocrit, total plasma protein, total white blood cell count, heterophils, lymphocytes, eosinophils, monocytes, and basophils were evaluated.

Assessment of hepatic, renal, and metabolic function included measurements of Albumin (ALB), Total Protein (TP), Globulin (GLOB), Albumin/Globulin ratio (A/G), Aspartate Aminotransferase (AST), Total Bile Acids (TBA), Creatine Kinase (CK), Uric Acid (UA), Glucose (GLU), Calcium (Ca+), Phosphorus (P+), Potassium (K+), and Sodium (Na+), following the reference values established for the genus *Amazona* sp. (14).

### 2.4 Serological diagnosis

Detection of anti-*S. pullorum* and anti-*M. gallisepticum* antibodies was performed using the rapid serum agglutination (RSA) plate test, employing the commercial kits Pulor Test® and Myco-Galli Test® donated by INATA LAB LTDA (Figure 1B). This method was used as a screening test due to its high sensitivity, allowing the detection of low antigen titers.

The assays were performed according to the manufacturer’s recommendations. One drop (50 μL) of the specific antigen and one drop (50 μL) of blood sample were placed onto glass plates using a volumetric pipette and disposable tips. The serum and antigen were homogenized with stirring rods for 10 s and maintained at rest for 60 s to allow the reaction to occur, observing the presence or absence of agglutination.

The Rapid Serum Agglutination (RSA) test used in this study detects circulating agglutinating antibodies directed against the investigated pathogens rather than the pathogens themselves. The assay is based on the interaction between specific serum antibodies and standardized antigens, resulting in visible agglutination. Although the test does not differentiate immunoglobulin classes, the agglutination reaction is predominantly associated with IgM antibodies produced during the early stages of the humoral immune response. However, IgY antibodies may also contribute to positive reactions depending on the stage and duration of infection. Therefore, positive RSA results should be interpreted as evidence of previous exposure or immune response to the pathogen rather than direct confirmation of active infection.

Positive and negative controls were included in all reactions to ensure reliability and validation of the tests. Samples were considered positive when visible clump formation (agglutination) was observed (Figures 1C,D).

### 2.5 Statistical analysis

The data were organized in a digital spreadsheet. Subsequently, the hematological, leukogram, and biochemical results of animals reactive to *S. pullorum* and/or *M. gallisepticum* and non-reactive animals were subjected to normality analysis using the Shapiro–Wilk test (*p* > 0.05). Data were compared using one-way ANOVA, followed by Tukey’s *post hoc* test, or by the Kruskal–Wallis test followed by the Student–Newman–Keuls post hoc test, when appropriate. A significance level of 5% was adopted for all comparisons, and statistical analyses were performed using SPSS 23 for Mac (15, 16).

## 3 Results

For *M. gallisepticum*, seroreactivity was observed in 21.73% of the *A. aestiva* specimens (5/22; 95% CI: 7.4–41.3%) and in 14.3% of the *A. amazonica* individuals (1/7; 95% CI: 0.4–57.8%). Regarding *S. pullorum*, 36.36% (8/22; 95% CI: 16.3–56.6%), reactive birds were identified among *A. aestiva* specimens and 28.60% of the *A. amazonica* individuals (2/7; 95% CI: 3.7–71.0%), as shown in Table 1.

**Table 1:** Detailed physical evaluation of *Amazona aestiva* (*A.Ae*) and *Amazona amazonica* (*A.Am*) parrots rescued from wildlife trafficking in the state of Paraíba.

Individual	Weight (median)	Score	Muscle tone	Feather coloration	F.D	Reactive
A.Ae 1	0.415	2	Strong	C.A + darkened	5	*
A.Ae 2	0.370	3	Strong	C.A + darkened	4	*
A.Ae 3	0.319	2	Strong	C.A + darkened	4	*
A.Ae 4	0.274	1	Flaccid	C.A + darkened	2	*
A.Ae 5	0.310	3	Strong	C.A + darkened	1	*
A.Ae 6	0.319	2	Flaccid	C.A + darkened	5	*
A.Ae 7	0.338	2	Flaccid	C.A + darkened	4	*
A.Ae 8	0.303	3	Flaccid	Color alteration	3	*
A.Ae 9	0.335	3	Strong	Color alteration	2	+
A.Ae 10	0.303	3	Strong	C.A + darkened	1	+
A.Ae 11	0.393	4	Strong	C.A + darkened	2	+
A.Ae 12	0.301	2	Strong	Color alteration	4	+
A.Ae 13	0.292	2	Flaccid	Color alteration	4	+
A.Am 1	0.285	5	Strong	C.A + darkened	5	*
A.Am 2	0.307	3	Flaccid	C.A + darkened	4	*
A.Am 3	0.291	1	Strong	C.A + darkened	5	+

F.D, fat deposition; *, reactive for *salmonella pullorum*;+, reactive for *mycoplasma gallisepticum*; C.A, color alteration.

From a clinical perspective, all animals reactive to *S. pullorum* presented dehydration associated with fecal alterations, characterized by darkened and diarrheic feces. Individuals reactive to *M. gallisepticum* exhibited respiratory wheezing upon auscultation, involving both the upper and lower respiratory tract. Among these animals, two individuals presented dehydration, and one bird (A.A4) required more intensive supportive clinical intervention, indicating greater systemic impairment.

**Figure 2:**
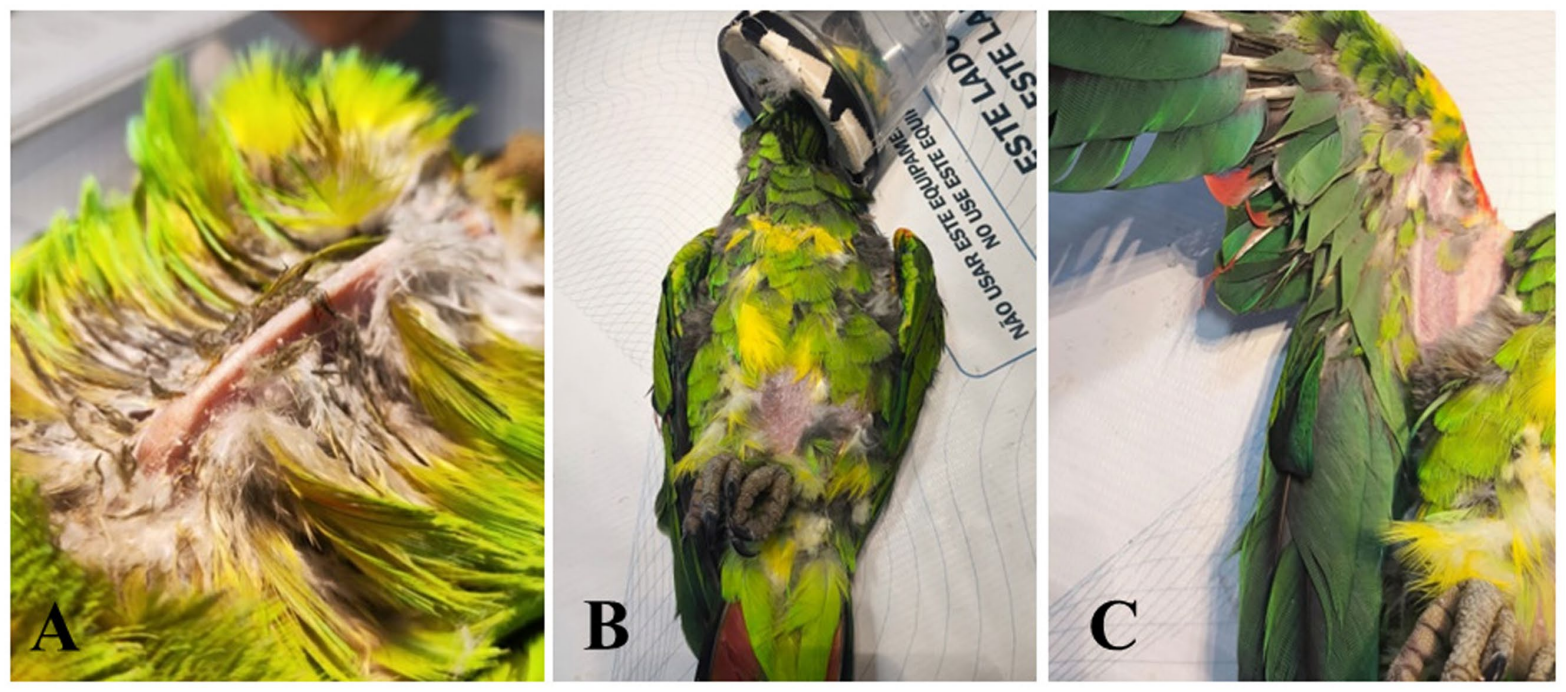
**(A)** Body condition score level 1 evaluation of individual *A.A4*. **(B)** Bird sedated by inhalation, showing the beginning of the physical examination, with observation of feather color alteration and apteria in the pectoral muscle region of *A. aestiva* rescued from wildlife trafficking. **(C)** Physical examination of the right wing of *A. aestiva* showing apteria of the medial remiges.

In the morphological evaluation, reactive *A. aestiva* individuals presented a median body weight of 0.319 kg, with an interquartile range (IQR) of 0.052 kg, whereas *A. amazonica* individuals showed a median of 0.291 kg and an IQR of 0.022 kg. Body condition score demonstrated a median of 2 (IQR = 1) for *A. aestiva* and a median of 3 (IQR = 4) for *A. amazonica* (Figure 2A).

Regarding muscle tone, 61.53% of *A. aestiva* individuals exhibited strong muscle tone and 38.46% presented flaccid tone, while in *A. amazonica*, 66.66% showed strong tone and 33.33% flaccid tone. Feather coloration assessment revealed relevant alterations, with 69.23% of *A. aestiva* individuals presenting feather darkening and 30.76% showing only color alteration. In reactive *A. amazonica* individuals, 100% presented color alteration associated with feather darkening (Figures 2B,C). Concerning body fat deposition, *A. aestiva* showed a median score of 4 (IQR = 2), whereas *A. amazonica* presented a median score of 5 (IQR = 1), as detailed in Table 1.

In the serum biochemical evaluation, it was found that 68.75% of the animals presented hypoalbuminemia, suggesting hepatic, inflammatory, or nutritional impairment. Regarding total proteins, 12.5% of the individuals showed values below the reference standards for the genus *Amazona*. On the other hand, 100% of the animals exhibited increased globulin levels, indicating activation of the immune system and systemic inflammatory response.

AST enzyme activity was elevated in 56.25% of the individuals, suggesting possible hepatocellular or muscular injury. Total bile acids (TBA) were elevated in 12.5% of the animals, all reactive to *S. pullorum*, reinforcing the possible hepatic involvement associated with infection. Creatine kinase (CK) levels were elevated in 75% of the individuals, consistent with muscular injury, recent restraint, or physiological stress. In addition, 50% of the animals presented reduced uric acid levels, possibly related to metabolic or hepatic alterations. In the glycemic evaluation, 25% of the individuals presented hypoglycemia and 25% hyperglycemia, demonstrating metabolic instability among the evaluated animals.

In the mineral metabolic evaluation, 56.25% of the animals presented hypercalcemia, while 6.25% exhibited hypocalcemia. In association, hypophosphatemia was observed in 43.75% of the individuals, and three animals simultaneously presented hypercalcemia and hypophosphatemia, indicating imbalance in calcium-phosphorus metabolism. Furthermore, 6.25% presented hyperphosphatemia. Regarding potassium levels, 18.25% of the animals showed hyperkalemia and 12.50% hypokalemia, with one case associated with hypercalcemia. Finally, 12.50% of the animals presented hyponatremia, suggesting electrolyte disturbances associated with the observed metabolic alterations.

In the hematological evaluation, 100% of the reactive animals presented heterophilia, while 81.25% exhibited lymphopenia, a pattern compatible with acute inflammatory response or chronic stress. All individuals reactive to *M. gallisepticum* showed increased total plasma protein (TPP) levels, and 80% of the animals reactive to *S. pullorum* also exhibited elevated TPP values, according to the reference values described by Carpenter and Harms (14), presented in Table 2.

**Table 2:** Biochemical and hematological evaluation of *A. aestiva* and *Amazona amazonica* parrots rescued from wildlife trafficking in the state of Paraíba (*p* < 0.05).

Parameter	*S. pullorum* (*n* = 10) mean	SD	*M. gallisepticum* (*n* = 6) mean	SD	Negative control (*n* = 13) mean	SD
Albumin (g/dL)	1.51	0.30	1.60	0.44	1.70	0.15
Total protein (g/dL)	3.65ᵃ	0.46	3.68	0.73	4.36ᵇ	0.75
Globulin (g/dL)*	2.15	0.36	2.08	0.48	2.66	0.79
Albumin/globulin ratio	0.72	0.19	0.79	0.23	0.69	0.18
Aspartate aminotransferase (U/L)	417.40	201.89	439.83	163.84	456.23	144.05
Total bile acids* (μmol/L)	40.15	34.95	30.20	16.52	38.82	26.04
Creatine kinase (U/L)*	1,064.94	1,026.56	449.67	234.74	519.31	455.84
Uric acid (mg/dL)*	2.26	1.97	2.35	1.78	1.74	2.11
Glucose (mg/dL)*	216.27ᵃ	44.90	276.93ᵇ	36.08	253.40	45.45
Calcium (mg/dL)*	30.34ᵃ	9.79	8.78ᵇ	0.50	8.60ᵇ	0.80
Phosphorus (mg/dL)*	3.77	1.58	3.55	1.16	4.47	1.57
Potassium (mmol/L)*	3.79	0.97	3.95	0.54	4.12	0.76
Sodium (mmol/L)*	130.88ᵃ	19.87	144.03ᵇ	1.29	140.82ᵃ	1.70
Hematocrit (%)*	48.60	8.13	47.00	10.94	51.54	6.95
Plasma protein	6.68	1.74	8.58	2.75	6.58	2.13
Total leukocytes (/μL)	12,621.14	5,489.27	8,242.13ᵃ	7,001.21	17,811.11ᵇ	6,472.72
Heterophils (%)	85.10	6.59	86.00	10.12	80.44	15.31
Lymphocytes (%)	11.70	6.13	11.33	11.24	8.89	5.23
Eosinophils (%)	1.71	0.76	1.00	0.00	1.83	0.75
Monocytes (%)	2.00	0.82	1.00	0.00	4.33	1.97
Basophils (%)	1.17	0.41	2.67	2.08	1.80	0.84

Different letters in the same row indicate statistically significant differences between groups (*p* < 0.05). *Parameters for which at least one group deviated from a normal distribution were analyzed using the Kruskal–Wallis test, followed by Student–Newman–Keuls post hoc analysis. The superscript letters indicate the results of the multiple comparison test. Values sharing the same letter are not significantly different, whereas values with different letters differ significantly at *p* < 0.05.

A significant reduction in total protein levels was observed in the group reactive to *S. pullorum* (3.65 g/dL; group “a”) when compared to the control group (4.36 g/dL; group “b”), indicating hypoproteinemia associated with salmonellosis, possibly related to chronic inflammatory processes or intestinal malabsorption, consistent with the fecal alterations clinically observed.

Regarding glucose levels, a statistically significant difference was observed among the groups, with the *M. gallisepticum*-reactive group presenting higher glycemic values (276.93 mg/dL) compared to the *S. pullorum*-reactive group (216.27 mg/dL), while the control group remained at intermediate values, suggesting that glycemic alterations were directly associated with the infectious agent involved.

Calcium presented the greatest statistical discrepancy, with the *S. pullorum*-reactive group exhibiting a mean value of 330.34 mg/dL, significantly higher (group “a”) than the *M. gallisepticum*-reactive group (8.78 mg/dL) and the control group (8.60 mg/dL), both classified within the same statistical group (“b”). This marked increase may reflect a severe metabolic response or analytical interference associated with infection.

Serum sodium levels were significantly higher in the *M. gallisepticum*-reactive group (144.03 mEq/L) compared to the *S. pullorum* group (130.88 mEq/L) and the control group (140.82 mEq/L). The *S. pullorum* and control groups presented statistically similar values, whereas the *M. gallisepticum* group showed the highest levels.

In the hematological profile, the *M. gallisepticum*-reactive group presented a significantly lower leukocyte count (8,242.13/μL; group “a”) compared to the control group (17,811.11/μL; group “b”), indicating a tendency toward relative leukopenia, possibly associated with the action of the pathogen on the immune system of reactive animals.

## 4 Discussion

The results obtained in this study demonstrate the occurrence of significant infectious agents in parrots of the genus Amazona rescued from wildlife trafficking. Seroreactivity to S. pullorum showed the highest overall prevalence among the investigated pathogens, whereas *M. gallisepticum* was detected less frequently, although it was consistently associated with clinical manifestations. In *Amazona aestiva*, the prevalence of *M. gallisepticum* was 21.73%, whereas in *A. amazonica* it was 14.3%. Regarding S. pullorum, a high frequency of reactive individuals was observed in both species, particularly in *A. amazonica* (28.6%), reinforcing the impact of wildlife trafficking as a predisposing factor for the dissemination of these pathogens. Adverse conditions such as stressful transportation, overcrowding, nutritional deficiencies, and the absence of sanitary protocols create a favorable environment for both intestinal colonization by S. pullorum and the establishment and persistence of respiratory infections caused by Mycoplasma, as widely described in the literature (17–19, 40).

The serological reactivity observed in the present study reflects the activation of the humoral immune response against S. pullorum and *M. gallisepticum*. In birds, IgM represents the first immunoglobulin produced following antigenic exposure and is characterized by its high agglutination capacity, making it the principal immunoglobulin detected by rapid serum agglutination tests. As the immune response progresses, IgY becomes the predominant circulating immunoglobulin, providing long-term systemic protection and indicating previous exposure to infectious agents (20, 21, 38).

The high frequency of seroreactivity observed for S. pullorum may therefore indicate both recent and previous exposure to the pathogen, especially considering the adverse conditions associated with wildlife trafficking, which favor pathogen transmission and maintenance (41). This interpretation is supported by the significant reduction in total protein levels observed in the S. pullorum-reactive group (3.65 ± 0.46 g/dL) compared with the control group (4.36 ± 0.75 g/dL), as well as by clinical evidence of enteric involvement, including diarrhea, dehydration, and impaired body condition. Together, these findings suggest that the detected antibodies were associated with a biological response compatible with gastrointestinal infection and systemic repercussions (39).

Similarly, the seroreactivity detected against *M. gallisepticum* may reflect ongoing or previous exposure to the pathogen. The significantly lower leukocyte counts observed in the reactive group (8,242.13 ± 7,001.21 cells/μL) compared with the control group (17,811.11 ± 6,472.72 cells/μL), combined with respiratory manifestations and significantly higher glucose concentrations (276.93 ± 36.08 mg/dL), suggest a physiological response compatible with chronic respiratory infection, stress, and persistent antigenic stimulation. In this context, the presence of agglutinating antibodies detected by RSA may reflect both the initial IgM-mediated response and the persistence of circulating IgY antibodies associated with prolonged immune stimulation. Consequently, positive serological reactions should be interpreted as indicators of host exposure and immune activation rather than direct evidence of active pathogen replication (20, 22, 23).

A high frequency of seroreactivity to S. pullorum was observed among the evaluated parrots, and this condition was accompanied by clinical alterations compatible with gastrointestinal impairment. Reactive animals presented diarrheic and darkened feces associated with dehydration, indicating active enteric involvement and possible systemic repercussions of the infection. These findings were accompanied by relevant biochemical alterations, particularly the statistically significant reduction in total protein levels in the S. pullorum-reactive group (3.65 ± 0.46 g/dL) compared with the control group (4.36 ± 0.75 g/dL), characterizing hypoproteinemia.

The reduction in total protein suggests impairment of protein homeostasis, possibly associated with chronic intestinal inflammation, gastrointestinal protein loss, and reduced nutrient absorption. These mechanisms are frequently described in birds affected by bacterial enteropathies, in which intestinal mucosal integrity is compromised, favoring both protein loss and secondary metabolic alterations (17, 24–26). Furthermore, the observed hypoproteinemia was associated with the clinical and morphological findings identified in reactive animals, including persistent fecal alterations, dehydration, and poorer body condition. Taken together, these results reinforce the hypothesis that S. pullorum infection promotes significant enteric and metabolic impairment capable of affecting the nutritional and physiological status of affected individuals.

In contrast, all individuals reactive to *M. gallisepticum* presented respiratory wheezing involving the lower respiratory tract, characterizing respiratory impairment compatible with mycoplasmosis. This clinical manifestation was accompanied by biochemical alterations, as the *M. gallisepticum*-reactive group exhibited significantly higher glucose concentrations than the S. pullorum-reactive group, while the control group presented intermediate values (23, 27). This statistical difference suggests that hyperglycemia may be associated with chronic stress responses, persistent systemic inflammation, and neuroendocrine activation induced by respiratory infection, phenomena widely described in birds affected by chronic mycoplasmosis (28).

Morphological evaluation revealed that although some animals presented body weights within the reference ranges described by Carpenter and Harms (14), there was considerable variation in body condition score, muscle tone, fat deposition, and feather coloration. The presence of low body condition scores, muscle flaccidity, and feather darkening suggests chronic nutritional and metabolic impairment, frequently associated with conditions imposed by wildlife trafficking. In animals reactive to S. pullorum, these alterations may be attributed to intestinal dysbiosis, enteric inflammation, and nutrient malabsorption, whereas in *M. gallisepticum*-reactive individuals they may reflect the systemic impact of chronic respiratory infection, considering the obligate intracellular nature of the pathogen (18, 29, 30).

Regarding the hematological profile, the data reinforces the association between the clinical, morphological, and laboratory findings observed in the evaluated animals. Particularly noteworthy was the fact that the *M. gallisepticum*-reactive group presented significantly lower leukocyte counts (8,242.13 ± 7,001.21 cells/μL) compared with the control group (17,811.11 ± 6,472.72 cells/μL), characterizing statistically significant relative leukopenia. This finding suggests possible pathogen-induced immunomodulation, potentially affecting cellular immune responses and compromising the animals’ ability to defend against secondary infections, especially in individuals subjected to adverse conditions associated with wildlife trafficking. Similar alterations have been reported in birds affected by chronic respiratory infections caused by Mycoplasma spp., in which persistent infection and prolonged antigenic stimulation may alter leukocyte dynamics and systemic inflammatory responses (22, 23, 31).

Although no statistically significant differences were observed in heterophil and lymphocyte percentages among groups, heterophils predominated in all evaluated groups, with mean values exceeding 80%, a pattern frequently associated with bacterial inflammatory responses and physiological stress in birds. Additionally, plasma total protein (PTP) values tended to be higher in *M. gallisepticum*-reactive animals (8.58 ± 2.75 g/dL) and in part of the S. pullorum-reactive individuals (6.68 ± 1.74 g/dL) compared with the control group (6.58 ± 2.13 g/dL). Although no statistical difference was detected for this parameter, these values may reflect dehydration, systemic inflammation, and persistent antigenic stimulation, as described by Arsenault et al. (32), Henry et al. (33), and Carpenter and Harms (14).

The biochemical alterations observed reinforce the systemic nature of these infections and their association with the clinical and laboratory findings. Particularly noteworthy was the statistically significant reduction in total protein levels in the S. pullorum-reactive group (3.65 ± 0.46 g/dL) compared with the control group (4.36 ± 0.75 g/dL), indicating hypoproteinemia compatible with chronic enteric inflammatory processes, intestinal protein loss, and impaired nutrient absorption (34).

Regarding glucose, a statistically significant difference was observed between the S. pullorum- and *M. gallisepticum*-reactive groups. Animals reactive to *M. gallisepticum* exhibited the highest glucose concentrations (276.93 ± 36.08 mg/dL), whereas S. pullorum-reactive individuals presented significantly lower concentrations (216.27 ± 44.90 mg/dL). The control group showed intermediate values (253.40 ± 45.45 mg/dL). These findings suggest that mycoplasmosis may be associated with a metabolic response compatible with chronic physiological stress and persistent inflammation, favoring increased circulating glucose levels. Conversely, the lower glucose concentrations observed in S. pullorum-reactive animals may reflect gastrointestinal impairment, including anorexia, reduced intestinal nutrient absorption, and increased metabolic demand associated with the infectious process. Similar findings have been reported by Arsenault et al. (32) and Ishfaq et al. (25), who related glycemic alterations to distinct pathophysiological responses triggered by bacterial infections in birds.

Calcium showed the greatest statistical discrepancy, with significantly elevated values in the S. pullorum-reactive group compared with both the *M. gallisepticum*-reactive and negative control groups, which shared the same statistical classification. This marked increase may reflect an exacerbated metabolic response, secondary hepatic or renal dysfunction, as well as possible analytical interference associated with the infectious process (35). This finding is consistent with the clinical and morphological alterations observed in animals reactive to salmonellosis, particularly dehydration, loss of body condition, and fecal abnormalities.

Serum sodium concentrations also differed significantly among groups, with the *M. gallisepticum*-reactive group presenting higher values than both the S. pullorum-reactive and negative control groups (25, 36). While the S. pullorum and control groups maintained statistically similar values, the *M. gallisepticum* group exhibited the highest sodium concentrations, suggesting hydroelectrolytic disturbances potentially associated with systemic inflammation, chronic stress, and prolonged respiratory alterations. These findings are consistent with dehydration, inflammation, and metabolic imbalance described in birds exposed to prolonged adverse conditions (25, 32, 37).

## 5 Conclusion

In an integrated perspective, the results indicate that parrots of the genus Amazona reactive to S. pullorum and *M. gallisepticum* presented multisystemic impairment involving morphological, clinical, biochemical, metabolic, and hematological alterations. These findings reflect the impact of the adverse conditions imposed by wildlife trafficking, which favor exposure to infectious agents and contribute to the establishment, maintenance, and progression of infectious processes in rescued birds. It is important to emphasize that the Rapid Serum Agglutination (RSA) test employed in this study detects circulating agglutinating antibodies rather than the pathogens themselves and does not differentiate immunoglobulin classes. Therefore, positive serological reactions should be interpreted as evidence of previous and/or current exposure associated with an immune response against S. pullorum and *M. gallisepticum*, rather than direct confirmation of active infection. Additional diagnostic methods, such as molecular detection or pathogen isolation, are required to confirm active infection and pathogen shedding. Nevertheless, the association between seroreactivity and the clinical, hematological, and biochemical alterations observed in the evaluated birds reinforces the biological and epidemiological relevance of these findings. The occurrence of seroreactive trafficked parrots highlights the need for rigorous sanitary surveillance, screening, diagnostic, and clinical management protocols in wildlife rehabilitation centers, aiming to protect animal health, support conservation programs, and reduce the risk of dissemination of infectious agents among susceptible avian populations.

## Data Availability

The original contributions presented in the study are included in the article/supplementary material, further inquiries can be directed to the corresponding author.
